# An unexpected acoustic indicator of positive emotions in horses

**DOI:** 10.1371/journal.pone.0197898

**Published:** 2018-07-11

**Authors:** Mathilde Stomp, Maël Leroux, Marjorie Cellier, Séverine Henry, Alban Lemasson, Martine Hausberger

**Affiliations:** 1 Université de Rennes 1, CNRS, UMR 6552 CNRS Ethologie Animale et Humaine, Université de Caen-Normandie, Station Biologique de Paimpont, Paimpont, France; 2 CNRS, UMR 6552 Ethologie animale et humaine, Université de Rennes 1, Université de Caen-Normandie, Rennes, France; Tierarztliche Hochschule Hannover, GERMANY

## Abstract

Indicators of positive emotions are still scarce and many proposed behavioural markers have proven ambiguous. Studies established a link between acoustic signals and emitter’s internal state, but few related to positive emotions and still fewer considered non-vocal sounds. One of them, the snort, is shared by several perrisodactyls and has been associated to positive contexts in these species. We hypothesized that this could be also the case in horses. In this species, there is a clear need for a thorough description of non-vocal acoustic signals (snorts, snores or blows are often used interchangeably) but overall this sound produced by nostrils during expiration has up to now been mostly considered as having a hygienic function. However, observations revealed that snorts were produced more in some individuals than in others, without relationship with air conditions. We observed 48 horses living in two “extreme” conditions: restricted conditions (single stall, low roughage diet) and naturalistic conditions (stable groups in pasture). The immediate place (e.g. stall/pasture) and the behavioural/postural (behaviour performed/ears positions) contexts of snort production were observed. We additionally performed an evaluation of the welfare state, using validated behavioural (e.g. stereotypies) and postural (e.g. overall ears positions) welfare indicators. The results show that 1) snort production was significantly associated with situations known to be positive for horses (e.g. feeding in pasture) and with a positive internal state (ears in forward or sidewards positions), 2) the riding school horses produced twice as many snorts when in pasture than in stall, 3) the naturalistic population emitted significantly more snorts than riding school ones in comparable contexts, 4) the frequency of snorts was negatively correlated with the composite total chronic stress score (TCSS, reflecting compromised welfare based on the horse’s rank on the different indicators): the lower the TCSS, the higher the snort rate. Snorts therefore appear as reliable indicators of positive emotions.

## Introduction

Assessing positive emotions in animals is important in the current developments on animal welfare [1]. It can help to define the situations perceived as more favourable by animals and to promote positive practices. Because emotions are short-lived (e.g. [2]), they correspond to an immediate internal state. However, there are still difficulties to measure them. Because animals in altered welfare are more prone to perceive negatively situations [3], finding reliable indicators of positive emotions may be a challenge in animals living in sub-optimal conditions. Indeed, emotions and their resulting behavioural and physiological components are often studied through or using experimental situations created and supposed to induce them, without checking the animals’ perception of the presumed positive situation itself. Thus physiological markers often give contradictory results. Heart rate frequency increases during food anticipation (a supposed positive condition: [4]), but decreases during a grooming simulation (interpreted as a relaxing positive event: [5,6]) in horses; cortisol concentration may increase or decrease during access to a food reward in pairs (pigs: [7]) and during positive interactions with humans (dogs: [8]), and its validity as a stress marker may vary between acute and chronic stress (e.g. [9,10]).

Some behavioural indicators of positive emotions have been proposed, such as the behaviours (minks: [11]; silver foxes: [12]) or vocalizations (pigs: [13]) expressed during the anticipation phase of a reward or meal, as revealing the expectation by animals of positive events. However, many of the behavioural components expressed in the anticipation context are ambiguous (e.g. yawning, stereotypic behaviours) (horses: [14,15]; elephants: [16]; cheetahs: [17]) and in any case reveal high intensity emotions reflected by agitation and rapid behavioural transitions [15,18]. Play behaviour has also been proposed and may reflect a transient positive state but it is an ambiguous indicator in adults as revealed by studies on horses and monkeys [19,20]. This behaviour may reflect a “coping mechanism” to unexpected events, learnt during situations inducing a loss of control caused by the individual itself [21]. It may also act in reducing social tensions [22]. Also, for most authors, only high intensity positive emotions are considered (e.g. social play: [1,18]; playful manual tickling: [23,24]). Thus it is rarer to find studies which mention indicators of lower intensity and less ambiguous positive emotions (e.g calm attention: [25]) and the search for clear visible indicators of positive emotions remains largely open.

Although there have been many studies relating acoustic signals and emotions, very few have broached the question of possible acoustic indicators of positive emotions (elephants: [26]; silver foxes: [27]; humans: [28]). Moreover, the few existing studies are often based on ambiguous states such as the anticipation of events (e.g. [29]). Non-vocal signals have rarely been investigated in this context but the few studies carried out on non-vocal sounds suggest a fairly close connection between acoustic production and internal state. Purring for example, considered by most authors as non-vocal sounds [30], is a well-known example of an acoustic cue mostly related to positive events, being produced in mammals in a ‘relaxed, friendly and probably reassuring/soothing mood’, playing the role of a contact signal produced in positive social situations and in absence of social tensions [31]. These observations were convergent in both natural and domestic conditions, including interactions between domestic felids and humans for instance [32]. Other signals, such as snorts, have been classified as reflecting positive (rhinos: [33], tapirs: [34]) or negative (tapirs: [35]) emotional states. Their acoustic structure is remarkable similar between species (including horses).

Horses produce vocal and non-vocal signals, including snorts (e.g. [36]). Several studies already showed that some of their vocalizations were enhanced in specific situations according to the valence associated [37,38]. Moreover, particular acoustic parameters of the whinnies have been proposed to encode either the level of arousal or the valence of emotions (e.g.[29,39,40]), making acoustic signals highly interesting markers of emotions. Although there is confusion in the terminology associated with non-vocal sounds in horses between studies (snorts, snores, blows being all the consequence of a “forceful exhalation through the nostrils”, the terms have been used interchangeably in many studies), leading to contradictory assumptions about their potential functions, snorts, as defined here (see further) have been mostly associated with a hygienic function of “clearing the nostrils of phlegm, flies or other irritants” [36,37]. However, anecdotal reports indicate that horses’ snorts are often heard during positive situational changes or, like in rhinos, while foraging (personal observations). In the present study, we hypothesized that snorts may, as a result of a mild positive excitation, be a behavioural reflection of a transient positive physiological change.

Animals living in good welfare conditions have more chances to experience a positive “mood” (e.g. horses: [41,42], pigs: [43]; poultry: [44,45]; sheep: [46]; rats: [47]; starlings: [3]). More recently, it was shown that horses living in “extreme welfare” conditions such as single stall housing, restricted roughage and intensive tight riding versus naturalistic conditions in stable groups in pastures and occasional relaxed leisure riding differed clearly in terms of cognitive biases hence in their chances to experience a positive emotion when the situation improves [48]: after having been trained to discriminate bucket positions with positive versus negative experiences, naturalistic-like horses tended to consider intermediate positions as positive (“optimistic” profile) contrary to horses living in restricted conditions (“pessimistic” profile). Therefore, in the present study, horses from three different populations, including these two extreme situations, were observed in their usual living conditions. The study included two aspects: 1) the immediate context and animal’s behaviour and posture at the time of snort production, which informed us of the valence of context and animal’s state; and 2) whether snort production was related to the animal’s chronic welfare state, as a potential modulator of positive emotions. We hypothesized that 1) snorts would be produced more when the animal was in a beneficial condition (i.e. pasture/stall, [49]) and 2) that horses in better welfare would be more prone to produce them than those with an altered welfare when in “improved” conditions.

## Materials and methods

### Ethical note

The experiments were carried out in 2016 in accordance with the European Parliament and the European Union Council relative to the animals’ protection used for scientific purposes directive 2010/63/UE and complied with the current French laws related to animal experimentation (decree n°2013–118 of 1 February 2013 and its five implementation orders (JO of 7 February 2013), integrated in the Code rural and the Code of the maritime fishing under n° R. 214–87 à R.214-137). The experiments made in this study did not enter in the scope of application of the European directive, thus in accordance with this directive and the current French laws, the following experiments did not require a request for authorization to experiment.

Private owners of the riding schools and of the leisure horses gave us the permission to conduct the study on their sites. Animal husbandry and care were under the management of the riding school staff and the leisure horses’ owners: the horses used in this experiment were not research animals.

### Terminology

**Fig 1:**
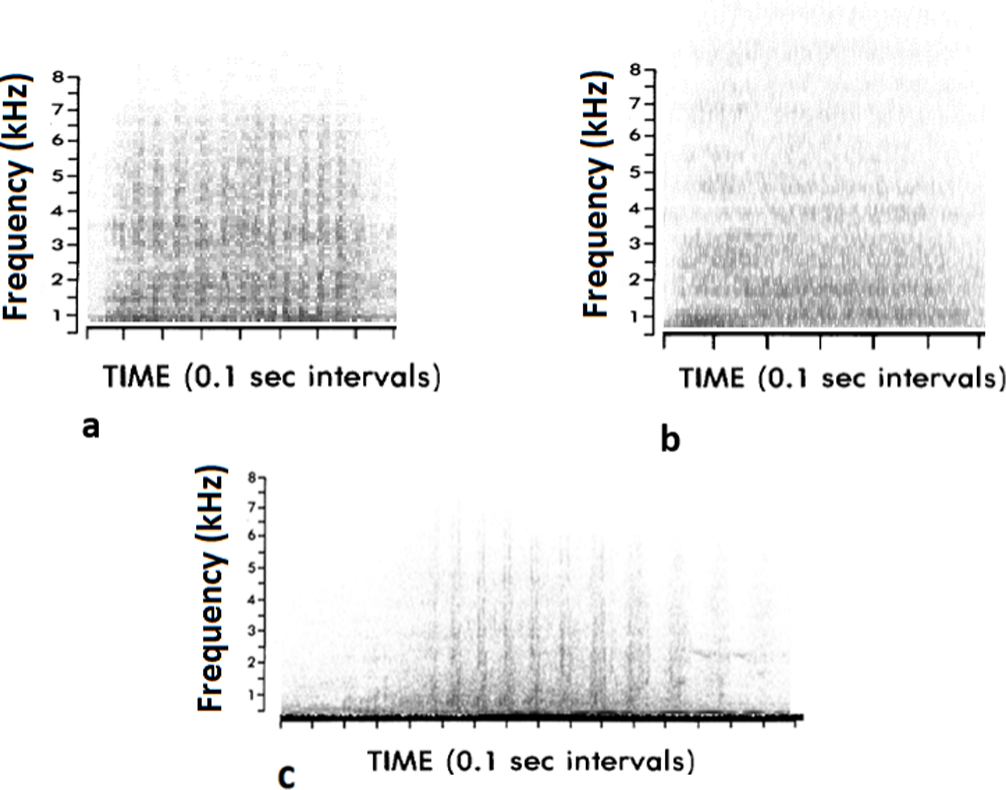
Spectrograms of a) a snore, b) a blow and c) a snort. The three sounds were extracted from the recordings performed in the present study.

Three different non-vocal sounds have been described in horses [50] (Fig 1). All of them are produced via the passage of the air through the nostrils. The snore is a very short raspy inhalation sound produced in a low alert context, such investigating a novel object or obstacle. It could also be produced prior to emitting a blow; the blow corresponds to a short very intense non-pulsed exhalation through the nostrils and is generally associated with vigilance/alarm postures (e.g. presence of a fear-inducing object in the surroundings); the **snort** corresponds to a more or less pulsed sound produced by nostril vibrations while expulsing the air, with a slightly longer duration in comparison to the blow. Since the description of the horse’s vocal repertoire is recent and still requires further investigation, there is still some confusion in the existing (mostly earlier) literature between these different sound categories (*e*.*g*. blows called snorts by several authors [51–53]). The confusion (of terminology) between snorts on one hand and snores and blows, which clearly reflect high fear responses [50] on the other hand, particularly in studies on emotionality and fear (e.g.[54–58]), shows how it is important to clearly define sounds on the basis of their acoustic structure in order to understand their causality and function. Clear examples showing the structural differences have been given by Waring [50] and Kiley [37] but no thorough description of their acoustic properties has been done yet. However, all agree that the distinction by ear is easy, only the choice of terminology varies according to authors.

In the present study we will consider a “snort” as a more or less pulsed broad-band sound of forceful exhalation through the nostrils, produced mouth closed (Fig 1).

### Subjects and management conditions

This study was conducted in Brittany (France) on a total of 48 individuals, 18 females, 25 geldings and 5 stallions, from various breeds (18 breeds represented, as well as unregistered or mixed-breed horses; 31.2% of French Saddlebreds) and ages (4 to 25 years). It involved four populations of horses (4 sites) (Table 1). On each site, only the horses that had been living on the same site and in these same conditions for at least one year have been observed. Moreover, only horses without known or visible respiratory problems have been considered.

**Table 1:** Characteristics of the living conditions by population. The time spent in individual stall and pasture was calculated considering a classical working day starting from 7am and ending at 7pm. The time spent in pasture was determined after an entire week of observations during the same time slot. Social instability may trigger more aggressiveness [76], “English” classical riding practices (e.g. short reins) have been linked to back disorder [54].

Population	Abbreviation	Number of individuals	Time spent in individual stall	Time spent in pasture	Social stability	Equitation
Riding School A	RSA	19	>90% of the time	1 to 4 hours per day	Unstable partners	English riding style
Riding School B	RSB	18	50% of the time	6 hours per day	Stable partners	English riding style
Naturalistic group	NC	11	Never in stall	Full time	Stable partners	Leisure

**Fig 2:**
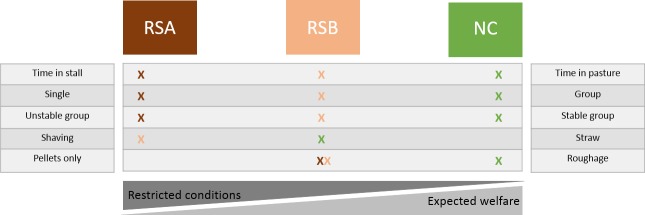
Expected welfare state according to the different living conditions’ characteristics observed [61–71]. Crosses correspond to the living conditions observed in each populations; cross colour corresponds to the population colour.

The first two populations were living in two riding schools (Riding School A (“RSA”): N = 19, 5 mares and 14 geldings, 4 to 15 years old, X +SE = 10.5+3.0 (the age of one of the horses was unknown); Riding School B (“RSB”): N = 18, 9 mares and 9 geldings, 5 to 22 years old, X +SE = 13+4.8). Both riding schools were characterized by restricted housing conditions (Fig 2): horses were kept in 3x3 m individual stalls in a barn (with door openings and grids in the wall allowing visual contact with conspecifics), fed industrial pellets twice a day (in the morning: 9:30 am in RSA, 8 am in RSB and in the evening: 6pm in RSA and 7pm in RSB) and hay (6-7kg) once a day (9am), and were working in riding lessons (including being ridden by riders from beginner to experienced) for 4–12 hours per week under the supervision of a riding teacher. The horses were ridden with typical English riding style (see also [6,59,60]). There were some differences though in the management practices between both facilities: the horses in RSA went out to paddocks (with grass) every day from one to four hours per day with variable social partners, while those in RSB went out as stable groups (from 2 to 11 individuals) into pastures (with grass) for about 6 hours per day every day. Another difference was that the stalls in RSA were straw bedded while they were shavings-bedded in RSB.

The other populations consisted in five different groups of leisure horses kept under naturalistic conditions year-round on two sites: social stable groups of 2–4 individuals (two family groups, one only-mares group, one all-male group, one gelding-mare group) in 1–2 ha natural pastures, fed grass and hay *ad libitum* during winter (no industrial pellets) and used for occasional outdoor relaxed leisure riding *(i*.*e*. low hands and long reins; see also [48,59]). One group belonged to the University of Rennes (N = 3, 3 stallions, from 5 to 16 years old, X +SE = 8.6±6.3) and the four other groups to a private owner (N = 8, 2 stallions, 2 geldings and 4 mares, from 12 to 25 years old, X +SE = 17.8±4.6). Given that no differences were found between groups in terms of age, snort rates, body conditions and welfare parameters (Mann Whitney tests, p>0.05,S1 Table), data were pooled in all subsequent analyses and these groups were merged under the name of “Naturalistic group” (“NC”) (Group NC: N = 11, 5 stallions, 4 mares and 2 geldings, from 5 to 25 years old, X +SE = 15.3±6.4).

### Data collection

#### Snort production and immediate context

The snort rates produced by each individual were measured by one single experimenter (M.L.), blind to the data on welfare assessment, using 5 min—Focal behavioural samples [72]. Horses from riding schools were observed both in stalls and in pastures while the leisure horses were only observed in pastures. The context (stall/pasture) and the assumed internal state (as expressed by behaviour/posture) of the animal at the precise time of production were examined.

In the stables, the experimenter performed 5 min—focal behavioural sampling, simultaneously on two neighbouring horses, focusing on the snort production. The experimenter followed a standardized route along the corridor separating the two rows of stalls and stopped at each pair of stalls encountered. The starting point of the route changed every day so that each pair of riding school horses was never observed at the same time of day over the entire study duration. For the outdoor observations, the experimenter followed again a standardized route that brought in to each different group. Again, sessions of 5 min—focal behavioural samples were performed facing the different groups (from one to four individuals maximum observed at the same time).

On average 12±3 focal behavioural samples were collected per horse per context (pasture and/or stall). One RSB horse could not be observed in the pasture condition. Thus when considering this context, samples were as follows: N_RSA_ = 19, N_RSB_ = 17, N_NC_ = 11.

Moreover, in order to determine the internal state of horses at the precise time of snort emission, the experimenter recorded the activity (e.g. eating, scanning the environment) and the ears position of the emitter associated to the snort production. The ears positon is known as an indicator of current internal state of the animal. In fact, in horses, backward ears position is commonly associated with negative emotional states, such as discomfort or pain (*e*.*g*. [73]; for a review: [74]) or during agonistic interactions (*e*.*g*. [50]), while forward or sidewards ear positions indicate rather attention and positive emotions [25,75,76]. Four positions were defined in accordance with previous studies (*e*.*g*.[60,77]):, forward (tip of the ear towards the front at an angle of more than 30° from the perpendicular), backward (tip of the ear towards the back at more than 30° from the perpendicular), asymmetrical (one specific direction for each ear) and sideward (auricles turning at 90°from the axial position—perpendicular to the head).

Snorts were sampled by ear but also thanks to a directional microphone (Sennheiser® K6/ME66), connected to a digital stereophonic portable recorder (PMD661 Marantz®, sampling rate 44100Hz, resolution 16 bits). The directional microphone was equipped with a wind for the outside context in order to soften background noises due to wind. Additionally, a microphone (Sony® ECM-T6) was used to record the experimenter’s voice, giving contextual and individuals’ information (see above).

In addition, we took the opportunity to record four leisure horses while they were going into a new pasture full of fresh resources. This situation allowed us to have a contrast situation as the one experienced by riding school horses with on one hand stall and on the other hand pasture. In the same way as in the outdoor condition, the first experimenter (M.L) measured snort rate production in several sessions of 5 min—Focal behavioural samples.

#### Baseline behavioural and postural observations

Finally, with the aim of studying the contrast of internal state expressed by the horses while snorting on one hand and during a baseline situation on the other hand, a second experimenter (M.S.) (blind to the snorts recording) made observations of 1) the horses’ activity according to a behavioural repertoire based on the typical horse ethogram [50] (S2 Table), and 2) the horses’ ears position, using an instantaneous scan sampling method [72] with 2 min intervals, in both contexts (stall/pasture). This enabled to have the time spent by the horses performing different activities as well as their ears positions associated while a basal situation. The latter observations were conducted in calm conditions (outside pellets feeding times, day off). Two sessions (1 in the morning, 1 in the afternoon) of 30 minutes per individual were conducted on one day. This operation was repeated one day after. Thus in total 62 scans of activity and ears positions per context were obtained per horse. These data allowed us to obtain a percentage of time spent doing each activity along with a percentage of each ears position expressed.

#### Welfare assessment

Welfare assessment was based on an array of validated behavioural, postural and health-related indicators. One observer (M.S.), blind to the data on the snorts, performed all the observations.

1. **Body assessment**
  Two measures were made once at the beginning of the study. First, body condition score (BCS) of each horse was evaluated according to Arnaud *et al*. [78]. The BCS results from an evaluation of the mass of fat deposits in five specific body locations (the upper edge of the neck, the whiter, the back of the shoulder, ribs, the tie of the tail) by palpation, by a visual assessment of seven anatomical sites (the upper edge of the neck, the whither, the back of the shoulder, the back line, ribs, the croup, the tie of the tail). According to a specific evaluation grid, each assessment received a score ranging from 0 (no fat deposits) to 5 (significant presence of fat): the average of the scores obtained defines the BCS of the horses ranging from 0 (emaciated) to 5 (obese).
  Neck shape has been shown to differ between populations independently of sex, age or breed, reflecting life conditions [79] and spine state [54, review in 64,65]. Precise measures of both neck shape and the spine state (through practitioner examination and EMGs evaluations) have shown that a hollow/flat neck reflects muscular tensions in different parts of the spine, while a round neck characterizes healthier backs [81]. Neck shape can easily estimated by looking at the angle formed by the segment linking the cervic-thoracic junction and the trapezium cervical ligament at C3 with the segment linking the trapezium ligament and the dorsal part of the atlas. According toLesimple *et al* [81]’s measures, the horses’ neck was classified as hollow, flat or round. Since flat and hollow necks tend to an altered spine state, the horses were classified as having a round or hollow/flat neck.
2. **Behavioural measures**
  - Stereotypic behaviours (SB, defined as “repetitive behaviours induced by frustration, repeated attempts to cope and/or brain dysfunction”; [82]) and other abnormal repetitive behaviours (ARB) have been shown to reflect inappropriate living conditions in horses (*e*.*g*. [69]) and chronic stress in a variety of species [83]. Stereotypic behaviours in horses are associated with lower cognitive abilities [84] and fertility [85]. Six sessions of observation (60 min in total per horse) were performed in the home stall. Observations were made during three time periods (twice per time period): 9–11 a.m., 2–5 p.m. and 30 min before the meals (favourable for observing abnormal repetitive movements (*e*.*g*. [14,86]), and at quiet times (outside teaching activities) with little disturbance by the routine procedures. Data recorded were the number of stereotypic behaviour and abnormal repetitive behaviours exhibited per individual (for 60 min).
    In the present study, the observer stood motionless at one point in the middle of the corridor at equal distance between the two rows of stalls so that she could see six horses at the same time. In stalls, horses generally performed SB/ARB only when their head was at the box door. The sampling was on an all-occurrence basis [53,71] which means that the behaviours concerned were scored each time they occurred together with the horse identity. SB and ARB were identified based on the list described by Lesimple *et al* [88] and Mills *et al* [69]. For a behaviour to be considered as SB/ARB, the behavioural sequence had to be repeated at least 3 times successively and observed 5 times, independently of the period of observation. Different types of these behaviours were observed (see further Table 1). Finally, we added up the number of stereotypic behaviours “SB” and abnormal repetitive behaviours “ARB” collected during all sessions for each horse.
  - Aggressiveness is a common expression of discomfort [87] or pain in horses as revealed by experimental studies [89,90] and it is used in a variety of welfare and pain scales (*e*.*g*.[73]; reviewed in [74]). Moreover, it has been shown that horses isolated in stall could become aggressive towards human, while working for instance [91]. In order to test individual aggressiveness, horses were submitted to three standardized human-animal relationship tests (*e*.*g*. [92]) in their familiar environment, performed in the same order for all horses:
    1. Sudden approach tests [93–95], where the experimenter, walking slowly along the corridor, appeared suddenly at the top part of the closed door of the box while the horse was feeding (hay, straw). The stalls were equipped with Dutch wooden-doors with the top and bottom divided, the bottom being solid and the top with wire grids. The horse’s first reaction was assessed as *non-aggressive* (*i*.*e*. looks at the experimenter with upright ears and approaches or does not show any evidence of directed attention toward the experimenter) or *aggressive* (*i*.*e*. looks at the experimenter with backward ears, and/or moves towards the experimenter with ears backward).
    2. A motionless person test (*e*.*g*. [92,96]), during which the experimenter entered the box and stood motionless with her back against the closed door, facing inwards and looking at the ground. The test lasted 1 minute. Data recorded were the total number of aggressive behaviours displayed by horses, including threats (threats always included ears backwards and could vary from simple threats, *i*.*e*. looking with ears laid back, threats to bite to threatening approaches) and real aggression (bites) (for more details, see [94]).
    3. An approach contact test where the experimenter was positioned at 1.5m from the subject and approached him perpendicularly, one step per second, up to the neck’s level. Then, she tried to touch the horse without forcing the contact. The horse was free to withdraw. The test ended when the experimenter succeeded in stroking the horse at least 2s, or after 3 unsuccessful trials. This test was conducted at right and left horses’ sides in a random order [94,97].
  We noted all agonistic behaviours (ears [94] laid back, threats, and attempts to bite) directed towards the experimenter during these three tests, performed in stall for the riding school horses, in the pasture for the leisure horses. Since no difference was found between the left and the right side (Wilcoxon test, N = 36, V = 31, p = 0.31), agonistic behaviours from both sides were pooled.
3. **Postural measures**
  - Ears positions were again measured for welfare assessment and thus recorded also outside the emission of snorts. In horses, backward ears position is commonly associated with negative emotional states, such as pain (*e*.*g*. [[73]]; for a review: [74]) and reflects chronic “mood” or state in horses [48,60] especially when recorded when horses are foraging [60]. The forward or sidewards positions are the ears position commonly observed while grazing [50]. The same four positions described above were considered (forward, backward, asymmetrical and sidewards). In order to have homogeneous conditions, the observer recorded ears positions only if the horse did not show any reaction (*i*.*e*. no change in behaviour) when she was observing, and in a single context: the horse had to be foraging on hay or straw or grass, head down as it has been shown to be the most reliable context ([98], and according to [60] and [48]). For the riding school horses, observations were made in the stables in calm conditions (outside pellets feeding times, day off, 2 to 5 pm, no wind). For the leisure horses, observations were made outdoors when horses were grazing, with the observer standing motionless outside the pasture (after at least 10min habituation). Ears position while feeding were scored using an instantaneous scan sampling method [72], with 2 min intervals. Two sessions (1 in the morning, 1 in the afternoon) of 30 minutes per context (stalls and pastures) and per individual were conducted on one day. This operation was repeated one day after. Thus in total 62 ears positions per context were obtained per horse. These data allowed us to obtain a percentage of each ears position during feeding for each horse.
  - The orientation towards the wall in the stall reflects a lack of interest towards the environment associated with potential health (e.g. anaemia [74]) or “psychological” [99]. In the present study we were only interested in orientations outside feeding time when animals were inactive. Therefore, horse’s orientation towards either a full wall (back wall, partitions) or outside (window or door top) was noted using instantaneous scan sampling method [72], with 2 min intervals. Two sessions (1 in the morning, 1 in the afternoon) of 30 minutes per individual were conducted on one day. This operation was repeated one day after. Thus in total 62 scans of orientation in the stall context were obtained per horse. These data allowed us to obtain a proportion of time (percentage of scans) spent with an orientation towards a full wall by individual outside feeding time.
  Given their living conditions all day long in pasture, this sampling could be not conducted for the leisure population.
4. **Overall welfare assessment**
  On the basis of the behavioural and postural data, a chronic stress score (TCSS) that reflects how much the chronic welfare state is altered, adapted from Hausberger *et al*. [19]’ and Henry *et al* [48]’s studies, was calculated for each horse. TCSS calculation consisted in ranking the horses according to 1) their number of aggressive responses during the three human-horse relationships tests, 2) the number of stereotypic behaviours displayed in 60 minutes of all-occurrence sampling sessions, 3) the percentage of scans spent with ears backwards while feeding, as well as 4) the percentage of scans spent facing the wall. For all of these variables, the higher the value obtained was, the poorer the welfare state was and the higher the rank attributed to the horse was. Ranks were added up between variables for each horse, such that at the end, the poorer the welfare of the horse the higher its TCSS. For instance, a horse that ranked 9^th^ lowest according to its number of aggressive responses, 8^th^ lowest according to its number of stereotypic behaviours occurrences, 5^th^ lowest according to its percentage of scans spent with ears backwards while feeding, and 8^th^ lowest according to its percentage of scans oriented towards wall got a TCSS of 30; which was higher, and consequently reflected a poorer welfare, than a TCSS of four obtained by a horse ranked 1^st^ lowest in all variables.
  Several composite scores were calculated according to the study population considered: one with all subjects combined (TCSS 1, N = 47), one with all horses in riding schools combined (TCSS_RS_, N = 37) and one for each facility (TCSS_RSA_, TCSS_RSB_ and TCSS_NC_).
  The TCSS score attributed when considering all three groups (TCSS 1) was based on the parameters 1) number of aggressive responses, 2) number of stereotypic behaviours and 3) percentage of scans spent with ears backwards while feeding, only since there was no possibility to evaluate the fourth parameter for the NC group. Since the pasture context was the only common context for the three populations observed, we considered the ears position while feeding based on pasture observations only when calculating TCSS1, considering all the horses together.
  Otherwise, the TCSS_NC_ specific to the naturalistic population was based on the behavioural parameters 1) number of aggressive responses and 2) number of stereotypic behaviours only, given that NC horses were living outdoor permanently and that no ears-back position during feeding time has been observed in pasture for this population.

#### Statistical analyses

As data were not normally distributed, we used nonparametric statistical tests [100].

As the number of 5min—Focal samples was not totally identical between subjects, each individual snorting rate was weighted considering the average number of focal samples (12 focal samples) done by the observer for all individuals on one hand, and the total number of focal samples realized by context for the individual on the other hand. Additionally, we calculated the inter- and intra- variability of snorts production among individuals using the coefficient of variation (CV = standard deviation/mean x 100) [101].

Mann-Whitney tests were used to compare TCSS scores between the two riding school populations, as well as their respective time spent ears backwards while feeding. Otherwise we used this test in order to compare the snort rates according to the type of neck.

We ran Wilcoxon tests in order to compare the snort rates depending on the emission context (pasture *vs* stall). Moreover the emitter’s ears positions while producing snorts were compared with that observed in the rest of the observations using a Wilcoxon test.

Kruskal-Wallis tests were performed to compare TCSS and BCS scores, and snort rates between populations followed by pairwise comparisons adjusted with Bonferroni correction.

Moreover, we ran chi squared tests for the purposes of comparing neck postures samples (number of horses) by populations.

Finally, in order to test the possible link between snorts production and welfare state, we conducted Spearman correlations. We investigated possible relations between snort rates and TCSS scores. First, we applied these correlations to: 1) all individuals pooled together, and 2) all riding school subjects. Possible specific links between snort emission and particular parameters of altered welfare were tested by running correlations with each altered welfare parameter (BCS scores, ranks) tested independently.

All statistical analyses have been conducted using R software 3.3.1 (alpha threshold set at 0.05).

## Results

560 snorts were recorded and all individuals produced some (0.75 to 12.8 per hour per individual). No differences according to sex (Mann-Whitney U-test, N_F_ = 18, N_M_ = 29, W = 292.5, p = 0.49), nor age (Kruskal-Wallis test, N1 = 6, N2 = 24, N3 = 16, X^2^ = 3.23, df = 2, p = 0.19) were found in regard to the total snort production (S3 Table).

### 1) Could snorts reflect current positive emotions?

#### 1.1) Contextual evidence: stall versus pasture

**Fig 3:**
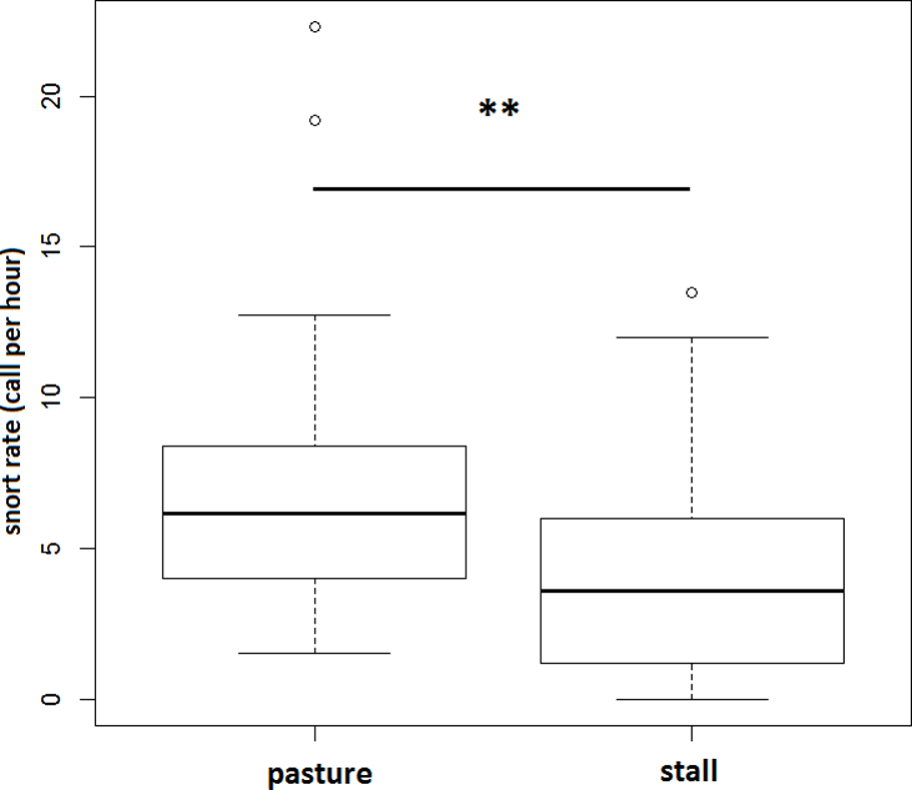
Snort rate according to two different contexts (stall and pasture).

In the two riding school populations, horses produced in average 5.66±3.32 snorts per hour. Snort rates clearly differed according to the context of production: overall subjects emitted significantly more snorts when they were in pasture than when they were in their individual stall (Wilcoxon test, N = 36, Z = 2.84, p = 0.004) (Fig 3). Indeed, eight horses did not produce any snort in stall. Thus, in total we recorded 454 snorts in riding school horses: 189 in stall (29 horses) and 265 in pasture (36 horses).

Otherwise the horses’ behavioural expression differed according to the context. The pasture context was less prone to the expression of aberrant behaviours since no SB/ARB nor withdrawn posture were observed when horses were outdoors contrary to the stall.

#### 1.2) Behavioural and postural evidence

1.2.1 Activities. Whether in stall or in pasture RS horses snorted mostly while eating (stall (hay): 67.3%, pasture (grass): 69.6% of snorts). They also produced more snorts during quiet observation when in stall (24.5%), and during slow exploratory walking when in pasture (20.3%). No snort was ever recorded while the animal performed a stereotypic behaviour, was facing the wall or was being aggressive (toward a human or another horse).

Similarly, the NC subjects performed snorts mostly while eating grass (68.9%), or in a slow exploratory walk (20.1%). In some cases, snorts were also produced just after rolling (5.4%).

1.2.2 Ears positions. *1*.*2*.*2*.*1 At the overall population level (outdoors)*. The time spent with the ears in forward/sidewards position measured during baseline observations was 92.8±16.9% when the horses were outside (pasture/paddock), but this proportion was significantly higher when only the moment of snorting was considered: 99.5±11.7% of the snorts were produced with the ears in this position (Wilcoxon test, N = 47, Z = 3.47, p<0.001). Logically, we found that horses expressed less ears backwards when producing snorts (μ_snorts_ = 0.19±1.32%) compared to the baseline level (μ_general_ = 4.58±2.23%) in pasture (Wilcoxon test, N = 47, Z = 2.99, p = 0.002).

*1*.*2*.*2*.*2 Stall context*: *The riding school populations*. In stalls, snorts were more often accompanied by forward/sidewards ears positions than in a general situation (μ_snorts_ = 92.7±20.8%; μ_general_ = 85.5±13.4%; Wilcoxon test, N = 29, Z = 2.51, p = 0.01) and less ears backward positions (μ_snorts_ = 4.5±18.4%) than in a general situation (μ_general_ = 8.2±9.4%) (Wilcoxon test, N = 29, Z = 2.84, p = 0.004).

**Fig 4:**
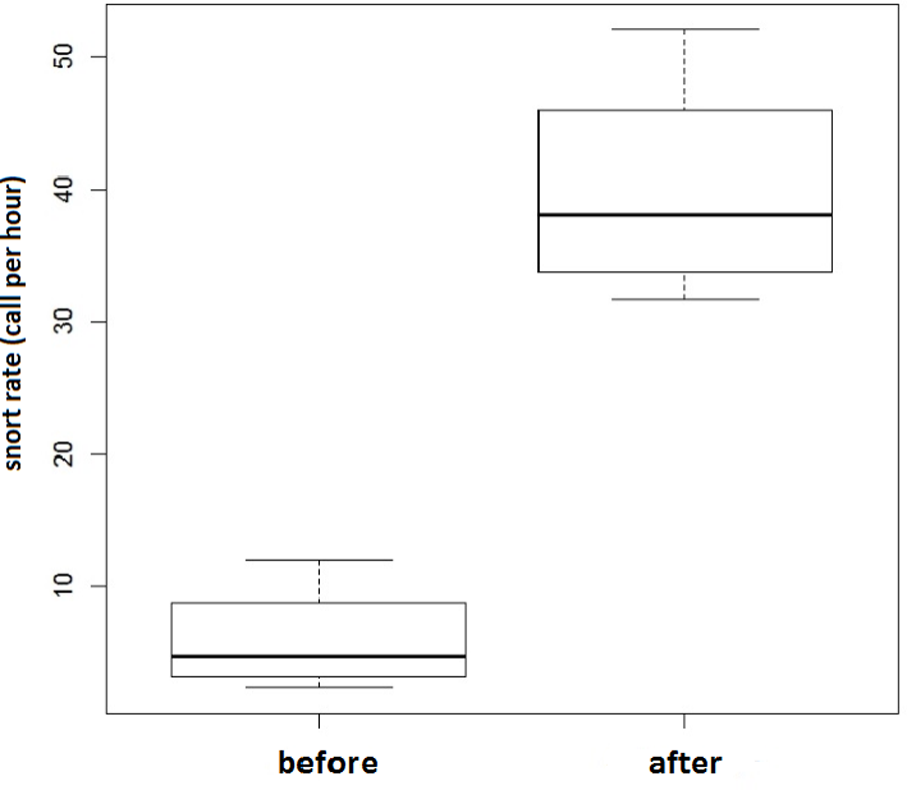
NC horses’ snorting rate observed just before and after being transferred from a low resource to a high resource (grass) pasture.

Finally, we had the opportunity to follow one group of the NC horses when it was moved to a new pasture with more feeding resources. The four horses produced up to ten times more snorts per hour than in the previous situation (μ_previous_ = 6±4.2; μ_new_ = 37.7±12.9) (Fig 4). Although anecdotal, this observation reinforces the idea of an association of snorts with positive emotions.

### 2) Could snort production be modulated by the welfare state?

#### 2.1 Comparison of snort production between populations

**Fig 5:**
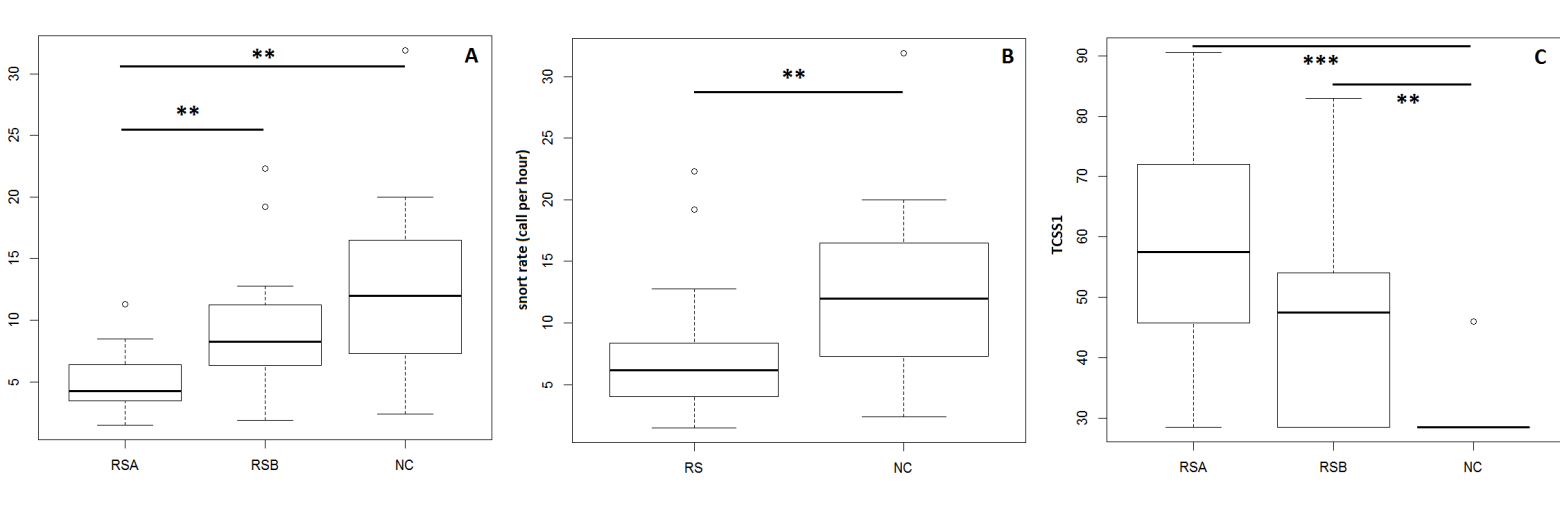
(A) The snorting rate observed in pasture according to the population of origin. (B) The snorting rate observed according to the type of the population, riding school horses and naturalistic horses. (C) The average of the TCSS 1 scores obtained by individuals according to the population of origin.

Considering only the pasture data, snort rates differed between the three populations (Kruskal Wallis test, N_NC_ = 11, N_RSA_ = 19, N_RSB_ = 17, chi-squared = 12.851, df = 2, p = 0.001). Post-hoc comparisons (including the Bonferroni corrections) showed that the snorting rate of the naturalistic horses was significantly higher than that of RSA (p = 0.008), but not RSB (p = 0.23) horses. Moreover, RSB horses produced more snorts than RSA horses (p = 0.009) (Fig 5A).

The NC population emitted significantly more snorts than the riding school horses when data were pooled for the two schools (Mann-Whitney U-test, N_NC_ = 11, N_RS_ = 36, W = 294, p = 0.01) with almost twice as many snorts (μ = 12.8±8.3; coefficients of intra-individual variation: 70.1% to 180.4%) than RSA and RSB horses taken together (μ = 7±4.6; coefficients of intra-individual variation: 110.8% to 519.6%) (Fig 5B). Intra-individual variability was lower in the NC population compared to the two RS populations indicating an apparent more stable state in these individuals experiencing the same environment (pasture all year long) over days. In contrast, we noticed a lower inter-individual variability in both riding school populations compared to that in NC population in pasture (coefficients of variation: inter: CV_NC_ = 65%, CV_RSA_ = 54.2%, CV_RSB_ = 57.2%). A possible group effect between naturalistic horses that experienced different types of resources according to their living pasture could occur. But we could not test it here since one sample was too small (N = 2) to be statistically relevant.

Interestingly, there was no difference between RSA and RSB snort rates when considering only the stall context (Mann Whitney tests, pasture: N_RSA_ = 19, N_RSB_ = 17, W = 72, p = 0.004; stall: N_RSA_ = 19, N_RSB_ = 18, W = 144.5, p = 0.42).

#### 2.2 Comparison of the welfare state between populations

Globally at the whole population level, horses’ Body Condition Scores (BCS) were between 2 and 4 (μ = 3±0.33). Out of the 48 individuals, 19 horses had a round neck whereas 29 showed a hollow/flat neck shape. 21 individuals (43.7%) performed at least one SB/ARB in stall (Table 2). Moreover, over the three HHR tests, 20 horses (42.5%) amongst which 18 from RSs population showed at least once an aggressive reaction towards the experimenter. Horses spent 4.5±11.7% of the time with ears in backward position while feeding in pasture. When in stall, the RS subjects were observed 22.1±24.6% of the feeding time with backward ears and 3±0.04% of the time facing a wall (N = 14 horses; 38.8%).

**Table 2:** SB/ARB observed during the study (see also Mills 2005).

SB/ARB behavioural description	Number of individuals involved
head tossing / nodding: vertical movements of head and neck	13
striking with forelimb: the horse hits the door or wall with one of its forelegs	4
box walking: repetitive tracing a route within the stable	1
cribbing / windsucking: the horse grasps a fixed object with its incisors, pulls backwards and draws air into its oesophagus	1
head movements (other than head tossing / nodding): movement of the head	5
tongue movements: movements of tongue, inside or outside the mouth	12
lips shivering: shivering of the lower lip	1
repetitive biting: biting of the same object in its environment (except the trough)	1
repetitive licking: licking of the same object in its environment (except the trough)	2
repetitive displacement of the saddle support on the stall's door	1

2.2.1 Body assessment. Horses’ body condition differed significantly between sites (Kruskal Wallis test, N_NC_ = 11, N_RSA_ = 19, N_RSB_ = 18, Kruskal-Wallis chi-squared = 8.82, df = 2, p = 0.01). NC horses had a higher BCS score (μ_NC_ = 3.13±0.45) than RSB horses (μ_RSB_ = 2.86±0.23) (post hoc pairwise comparisons, p = 0.038). By contrast, no significant difference was found between NC and RSA horses (μ_RSA_ = 3.07±0.30) (p = 0.73), nor between the two different RS groups although there was a trend for RSA horses to be slightly bigger (p = 0.06).

2.2.2 Postural measures. There were more horses with round necks (NC = 8, RSA = 5, RSB = 6) and less with hollow/flat necks (NC = 3, RSA = 14, RSB = 12) in the naturalistic population compared to both RS schools pooled (Chi squared test, N = 47, df = 1, X^2^ = 4.88, p = 0.027). More RSA horses had hollow/flat necks than round ones (Chi squared tests, X2 = 4,26, p<0.05).

Considering the ears positions recorded while feeding outside, RS horses spent 5.9±13.2% of this time with the ears backwards whereas NC horses were never observed feeding with the ears in this position (μ = 0±0%). When considering the RS horses only, some differences appeared: RSA horses spent more time feeding with ears backward in stall (RSA: 31.9±28%; RSB: 10.6±14.1%) (Mann Whitney test, N1 = 19, N2 = 18, W = 257, p = 0.008), but not in pasture: 6.4±11% of the time for RSA horses and 5.4±15.6% of the time for RSB ones (Mann-Whitney U test, N1 = 19, N2 = 17, W = 192, p = 0.29).

2.2.3 Behavioural measures. Clear signs of altered welfare were observed in both riding schools with ten types of SB/ARB (Table 2) observed and many subjects performing one or more (RS_A_ = 63.1%, RS_B_ = 44.4%) of them. Moreover, 66.6 and 31.5% of the RSA and RSB horses respectively displayed at least once an aggressive reaction towards the experimenter during the tests. In contrast, the leisure horses showed none of these abnormal and stereotypic behaviours. Moreover, only one aggressive behaviour was directed towards the experimenter by one NC horse.

2.2.4 Total chronic stress scores (TCSS). When comparing the TCSS1 scores obtained by individuals according to the three populations, it appeared that they significantly differed from each other (Kruskal Wallis test, N_NC_ = 11, N_RSA_ = 19, N_RSB_ = 17, H = 21.265, df = 2, p<0.0001) with NC subjects having a quasi-null TCSS1 score (post hoc pairwise comparisons: NC/RSA: p<0.0001; NC/RSB: p<0.001) while no significant difference was found between the TCSS 1 scores of the two riding schools populations (p = 0.27) (Fig 5C).

Moreover the TCSS_RS_ scores were lower in RSB than RSA: RSB individuals had a lower TCSS_RS_ score than the subjects studied in RSA (Mann Whitney test, N1 = 19, N2 = 17, W = 238, p = 0.015).

There was therefore a welfare gradient going from NC horses (best welfare) to RSB (intermediate) and then RSA (most altered welfare) (Fig 5C).

#### 2.3 Linking snort production to welfare assessment

Body assesment showed that, while there was no correlation between BCS scores and snort rates (Spearman correlation, N = 47, r = -0.039, p = 0.79), the latter tended to vary according to neck shape with twice as many snorts in horses with round necks (11.24±3.58) than in those with a hollow/flat neck (μ = 6.60±8.27) (Mann Whitney test, N_round_ = 18, N_flat/deep_ = 29, W = 346, p = 0.06).

**Fig 6:**
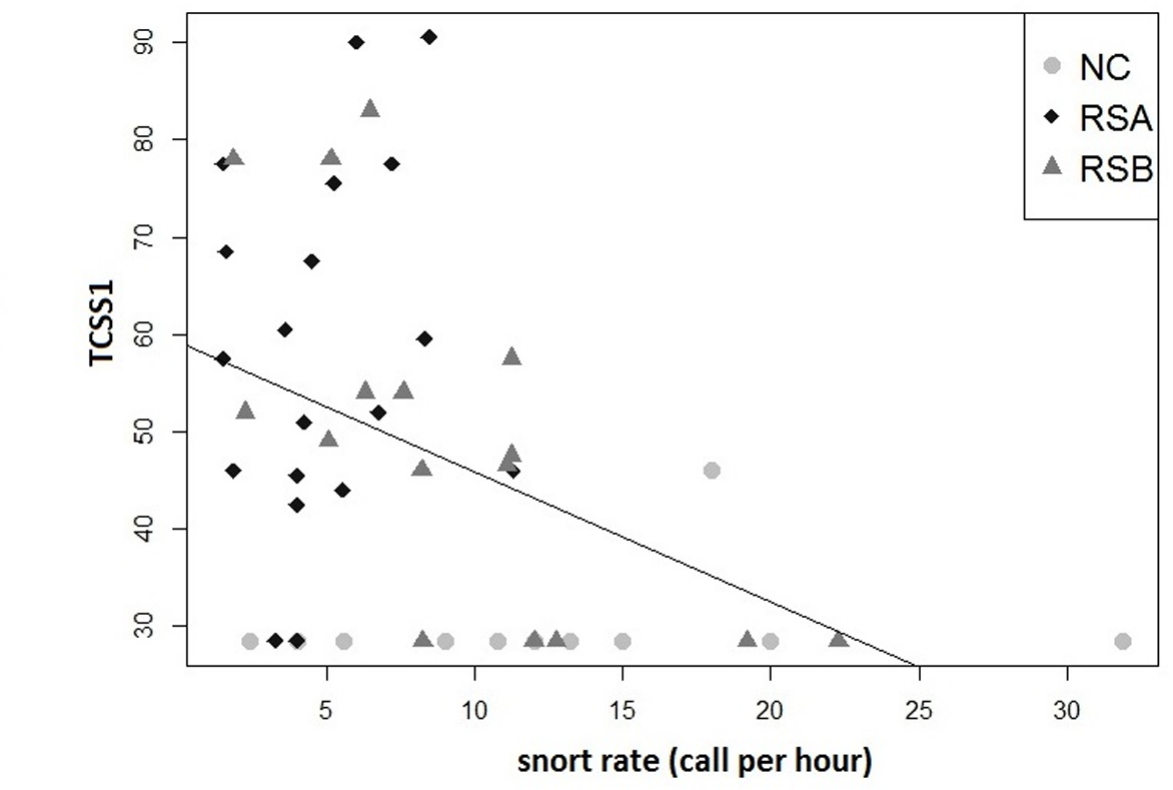
Correlation between the TCSS 1 score and the snorting rate of all individuals gathered.

Interestingly, we found a correlation between the snort production and the welfare scores. Indeed, snort rates collected in pasture (context common to all three populations) were negatively correlated to the TCSS1 (Spearman correlation, N = 47, r = -0.41, p = 0.003) (Fig 6).

**Fig 7:**
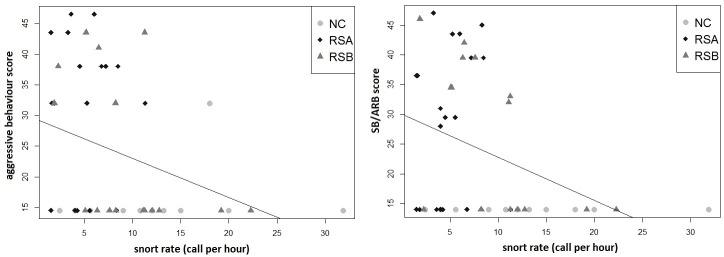
Correlation between snorting rate and particular TCSS1 score parameters: aggressive behaviours towards Human score (A), SB and ARB score (B).

Hence, the more horses emitted snorts, the more they were in a good welfare state. More precisely, the frequency of snorts produced in pasture was negatively correlated with the occurrence of aggressive behaviours during the human-horse relationship tests (Spearman correlation, N = 47, r = -0.33, p = 0.02) (Fig 7A) and the occurrence of SB/ARB (observed in stall) (N = 47, r = -0.32, p = 0.02). Again, the more horses emitted snorts, the less they were aggressive towards humans in the tests and the less they expressed SB/ARB behaviours (Fig 7B).

Moreover, the snort-welfare relationship was confirmed and strengthened when considering only the two RS populations: the lower the TCSS (combining postural and behavioural measures), the higher the snort rate (Spearman correlation, N = 36, r = -0.46, p = 0.004). This correlation remained significant when running the comparison with only the data collected in pasture (N = 36, r = -0.44, p = 0.007), however it was less true (just a trend) with data collected in stalls (N = 37, r = -0.28, p = 0.08). Interestingly though, the snort rate in stalls was negatively correlated with the occurrence of aggressive behaviour towards human during the tests (N = 37, r = -0.44, p = 0.005) while the snort rate in pasture was negatively correlated with the percentage of time spent facing the wall in stall (Spearman correlation, N = 36, r = -0.42, p = 0.01).

## Discussion

This study, which aimed at testing the potential interest of snorts as indicators of positive emotions, has revealed that 1) snort production is associated with more positive contexts (in pasture, while feeding) and states (with ears on forward position), 2) is less frequent in horses showing an altered welfare. These results provide a potential important tool as snorts appear as a possible reliable indicator of positive emotions which could help identify situations appreciated by horses. The results also indicate that positive emotions may not be measurable in unfavourable conditions independently of the animal chronic welfare state (ex: stall). However, animals in a better welfare state, appear to be more prone to produce them when the situation improves.

Given this array of results, it is clear that snorts cannot be merely considered as having a simple hygienic function of clearing the nostrils, expressed during no particular context nor in a specific arousal state [36,37,50]. Air conditions/dust cannot explain the present results as different horses in the same air conditions could differ in terms of snort production (e.g. correlation with welfare score). Moreover, one would then expect more snorts to be produced in the more dusty environment (ex: shavings of RSB’s litter, dry hay) of the stall than in pasture, which is the exact reverse of what was observed in our study. Using preference tests, Lee *et al* [102] and SØndergaard *et al* [49] demonstrated that horses were more motivated to be in pastures rather than in stalls, especially if other horses were also present outdoors. Being positively influenced by this context is therefore a better explanation for the higher frequency of snorts in pastures. Moreover, snort production was also associated with more positive posture (ears forwards/sidewards, reflecting a more positive state, *e*.*g*. [50]) and preferred activities such as foraging (grass or hay), or in a quiet observation, two activities reflecting relaxed quiet states [50]. The fact that horses living in different pastures produced different snort rates could be due to the different resources in their respective pasture. It would be interesting to confirm the transient aspect of snort production by comparing outdoor observations in good versus adverse weather conditions for example.

The chronic welfare state influences snort production. As already demonstrated in earlier studies, restrictions in spatial and social conditions at least play an active role in determining the welfare state of domestic horses (*e*.*g*.[60,62,71,86]), as can be the case also for working conditions (*e*.*g*. [57,59,80]). Here we found a welfare gradient from the stable outdoors leisure horses to the most restricted conditions of one riding school where horses went only occasionally out in unstable groups. Interestingly, the same gradient was found in snort rates recorded in pastures: the more altered the welfare is, the lower the snort rate. This was true also when particular parameters, such as aggressiveness towards humans or time spent in stall facing the wall, were considered. Horses with less dorsal problems, as reflected by their neck shape [81], produced also more snorts. Horses in good welfare state are more prone to experience positive emotions when given the chance [48] which does explain that they produce more snorts. However, snorts do indicate transient and not chronic states as no such differences were observed in the unfavourable condition of the single stall housing, known to lead to an altered welfare state (e.g.[60,71]).

Snorts might therefore be a very useful indicator of positive emotions. The search for acoustic markers of emotions is not new (*e*.*g*. horses: [38,58]; pigs: [103]; chicks: [104]) and many studies have assessed the intensity of the situation by counting distress or contact calls (*e*.*g* horses: [105]; pigs: [13]; sheep and cattle: [106]). Most acoustic studies have thus concentrated on emotions’ intensity (review in [107]), of a very high level for most of them. Conversely, very few studies have been demonstrating acoustic variations related to emotional valence. In horses, Briefer *et al* [29] proposed that whinnies’ duration and whinnies’ higher fundamental frequency (G0) encode valence; in rats, 50 kHz vocalizations have been proposed as markers of positive emotions [108]. Otherwise, studies on non-vocal signals are still scarce. Purrs have been broadly studied in felids and associated to positive social situations (review in [31]). This acoustic signal has been also reported in other species such as several primates: mouse lemurs [109], uakaris [110], ring tailed lemurs [111], mostly during positive social interactions (*e*.*g*. grooming) with a possible function of increasing tolerance; or during mother-infant interactions (*e*.*g*. prior to the beginning of suckling in squirrel monkeys: [112]). Snorts have been previously described in some perissodactyls during positive contexts, either while foraging (rhinos:[33,113]) or again during short distance communication between a mother and her young (while licking) as “an appeasement call” (tapirs: [34]). Kiley [37], in a detailed study on Ungulates vocalisations, proposed an acoustic gradient following an excitement gradient which included both non-vocal and vocal sounds. In this gradient from sigh to long whinny (high excitement), she proposed that the snort was closed to nickers (a vocalization associated with positive anticipation (Przewalski’s horses: [114]; horses: [50]) and noted that “the closed mouth nicker and whinnies of the horse could be regarded as a further development of a voiced snort”. Thus snort would embody a signal reflecting positive emotions of low intensity. Analogically, recently a neurophysiological study proposed that sighs could be partly the result of a normal breath transformed by an emotional input in rats [115]. Indeed, a high rate of sighs have been correlated with a release phase created after a sequence of electric shocks in this species [116]. Authors suggested that it would be used as a social calming signal to synchronise the emotional state of the group of conspecifics. Sigh has been also mentioned as a reflect of a relaxation state in humans [117].

To conclude, this study calls snort function into question. We propose that it would be indicating of a relaxation phase associated with positive emotions of low intensity and thus expressed even more by horses in a chronic good welfare state. However, our study does not totally allow to rule out the sanitary function of snort, since dust differences present in stall and pasture contexts have not been examined in details, but the results show that this is unlikely to explain differences between individual horses. Further researches involving dusts’ rate measures would be helpful to entirely determine the relative importance of this factor in snorts expression. It would also be important that a proper repertoire of the non-vocal sounds is promoted and becomes consensual between non-acoustic researchers on horses’ emotions to avoid confusions between studies and interpretations. Finally, observations during working circumstances would be needed where breathing constraints due to exercise and emotions are mingling. In any case, these results lead us to believe that acoustic signals constitute a potential marker of positive emotions, suggesting a new breakthrough in research evaluation of positive emotional states in horses.

## Supporting information

S1 Table Original p values obtained after running the Mann Whitney tests comparing NC1 and NC2 data. (PDF)

S2 Table The behavioural repertoire used for the study, adapted from a typical horse ethogram (McDonnell, 2003; Waring, 2003). (PDF)

S3 Table Individuals’s data collected in the present study. (PDF)
