# Supplementary material for: An unexpected acoustic indicator of positive emotions in horses
Source: PLoS One. 2018 Jul 11;13(7):e0197898. doi: 10.1371/journal.pone.0197898 (PMC6040684; doi:10.1371/journal.pone.0197898)
Supplement: S1 Table — (PDF) [file pone.0197898.s001.pdf]

Appendix 1

Table 1: Original p values obtained after running the Mann Whitney tests comparing NC1 and NC2 data.

variable tested	Mann Whitney	p value
age	W=21	p=0.08064
snort	W=6	p=0.2788
body condition	W=8	p=0.4296
SB/ARB	W=12	p=1
agressive reactions	W=8	p=0.153
ears backwards	W=12	p=1
