# Supplementary material for: An unexpected acoustic indicator of positive emotions in horses
Source: PLoS One. 2018 Jul 11;13(7):e0197898. doi: 10.1371/journal.pone.0197898 (PMC6040684; doi:10.1371/journal.pone.0197898)
Supplement: S2 Table — (PDF) [file pone.0197898.s002.pdf]

Appendix 2

Table 1: The behavioural repertoire used for the study, adapted from a typical horse ethogram (McDonnell, 2003; Waring, 2003).

BEHAVIOUR	DESCRIPTION
**Maintenance behaviour**	
Eat	Ingest grassy vegetation; hay with the lips and tongue. Food is gathered into the mouth and chewed.
Drink	Ingest water, typically by using lips at the surface of water, and drawing water with sucking action and swallowing.
Urinate	Expelling of urine through the urethra (males) or the urethra and vulva (females).
Defecate	Expelling of faeces through the anus.
In locomotion	Voluntary movement of the whole body on land. Includes exploratory walk, walk, trot, canter.
Rest standing	Standing inactive in a relaxed posture, usually with head slightly lowered, eyes partly or nearly closed, and often bearing weight on three legs (one hind leg slightly flexed).
Sleep standing	With eyes closed and head lowered below the back, light sleep in a standing position.
Rest recumbent	Rest or sleep while lying down with head up or with legs and head outstretched.
Yawn	Deep, long inhalation with mouth widely opened, with jaws either directly opposed or moved from side to side.
Self-grooming	Any comfort behaviour including licking, nibbling, scratching, rubbing, rolling, shaking and skin twitching and tail switching.
Allogrooming	Interactive nibbling between two individuals. The two partners usually face each other, standing so that one shoulder is close to the corresponding shoulder of the partner.
Investigation	Exposing the various sensory modalities to one stimuli. Includes sniffing, licking, manipulating with open mouth and pawing.
Monitoring the environment	Includes head movements to scan the environment and gazing towards the environment while the body remains immobile.
Look at an object	Gazing towards a specific object, without any head movements.
Look at a congener	Gazing towards a congener, without any head movements.
Look at a human	Gazing towards a human, without any head movements.

In vigilance	The neck is elevated, ears are upraised forward, oriented towards the stimulus as the head, nostrils sometimes slightly dilated.
Produce a non-vocal sound	Any unvoiced sound production (snort, snore, blow, grunt).
**Communicative behaviour**	
Vocalize	Any production involving the vocal cords (whinny, nicker, squeal).
**Social behaviour**	
Socio-negative behaviour	Aggressive display towards a congener or a human including threat, bite, strike and kick.
Socio-positive behaviour	Positive social display towards a congener or a human ranging from a positive approach with eras forwards to tactile interactions as nuzzling, licking, sniffing.
**Aberrant Behaviour**	
Weaving	Obvious lateral movement of head, neck, forequarters and sometimes hindquarters.
Cribbing	The horse grasps a fixed object with its incisors, pulls backwards and draws air into its oesophagus.
Head tossing/nodding	Vertical movements of head and neck.
Striking with forelimb	The horse hits the door or wall with one of its forelegs.
Box walking	Repetitive tracing a route within the stable.
Compulsive licking	Licking of the same object in its environment (except the trough).
Compulsive biting	Biting of the same object in its environment (except the trough).
Head movements (other than head tossing / nodding)	Movement of the head.
Vacuum threats	The horse express threat sequences (kicking, biting) alone in its box.
Mouth open	The horse keeps its mouth open with a lateral movement of its neck.
Teeth rubbing	Rubbing teeth on the upper part of the door.
Lips movements	Clapping of lips.
Tongue movements	Movements of tongue, inside or outside the mouth.
