# Supplementary material for: An unexpected acoustic indicator of positive emotions in horses
Source: PLoS One. 2018 Jul 11;13(7):e0197898. doi: 10.1371/journal.pone.0197898 (PMC6040684; doi:10.1371/journal.pone.0197898)
Supplement: S3 Table — (PDF) [file pone.0197898.s003.pdf]

Individual	Population	Sex	Age	Breed
Bul	NC	gelding	5	Recognized origin
Bounty	NC	gelding	5	Recognized origin
Momo	NC	gelding	16	Pottok
Pepito	NC	gelding	12	Recognized origin
Haribo	NC	gelding	21	Unrecognized origin
Kansou	NC	mare	18	Unrecognized origin
Julie	NC	mare	19	Unrecognized origin
Whinney	NC	stallion	25	Palomino
Quassie	NC	mare	12	Unrecognized origin
Hookah	NC	stallion	21	Unrecognized origin
Paloma	NC	mare	15	Unrecognized origin
Cooper	RSA	gelding	4	Conemara
Artiste	RSA	gelding	6	French Saddle
Man	RSA	gelding	15	French Saddle
Talisman	RSA	gelding	9	French Saddle
Centurion	RSA	gelding	9	German
Quartz	RSA	gelding	12	Welsh
Orion	RSA	gelding	14	English thoroughbred
Stevens	RSA	gelding	7	English thoroughbred
Ram Dam	RSA	gelding	11	English thoroughbred
Lady	RSA	mare	8	English thoroughbred
Quaïnah	RSA	mare	NA	Draught Horse
Taka	RSA	mare	12	English thoroughbred
One girl	RSA	mare	14	English thoroughbred
Palo	RSA	gelding	9	Irish
Royal	RSA	gelding	11	French Saddle Pony
Nectar	RSA	gelding	15	Landais
Ricky	RSA	gelding	11	Conemara
Quitto	RSA	gelding	12	French Saddle Pony
Sisi	RSA	mare	10	French Saddle Pony
Phaistos	RSB	gelding	13	French Saddle
Asterix	RSB	gelding	6	French Saddle and Belgish mixing
Balzan	RSB	gelding	5	New forest
Ty time	RSB	gelding	9	Irisih sport horse
Manu	RSB	gelding	16	French Saddle
Tempo	RSB	gelding	9	French Saddle
Loft	RSB	gelding	17	French Saddle
Laszlog	RSB	gelding	19	Westfalen
Menecerf	RSB	gelding	16	French Saddle
Qualine	RSB	mare	12	French Saddle Pony
Nouba	RSB	mare	15	French Saddle
Magie	RSB	mare	16	French Saddle
Saphira	RSB	mare	10	Irish Cob
Gorgona	RSB	mare	22	American/Irish thoroughbred
Ratina	RSB	mare	11	French Saddle
La plus	RSB	mare	9	Zangersheide
Iraty	RSB	mare	20	Trotteur français
Saouré	RSB	mare	10	Saddle Horse

Individual	Population	Neck shape	BCS
Bul	NC	round	3
Bounty	NC	round	3,5
Momo	NC	round	3,5
Pepito	NC	round	3,5
Haribo	NC	flat	3
Kansou	NC	round	3,5
Julie	NC	round	3
Whinney	NC	flat	2
Quassie	NC	flat	3,5
Hookah	NC	round	3
Paloma	NC	round	3
Cooper	RSA	round	3
Artiste	RSA	round	3,5
Man	RSA	flat	3
Talisman	RSA	flat	3,5
Centurion	RSA	round	3
Quartz	RSA	flat	3
Orion	RSA	flat	3
Stevens	RSA	flat	3
Ram Dam	RSA	round	2,5
Lady	RSA	flat	3
Quaïnah	RSA	round	4
Taka	RSA	flat	3
One girl	RSA	hollow	3
Palo	RSA	flat	3
Royal	RSA	flat	3
Nectar	RSA	hollow	3
Ricky	RSA	flat	3
Quitto	RSA	hollow	3
Sisi	RSA	flat	3
Phaistos	RSB	flat	2,5
Asterix	RSB	hollow	2,5
Balzan	RSB	hollow	3
Ty time	RSB	flat	2,5
Manu	RSB	flat	2,5
Tempo	RSB	round	3
Loft	RSB	round	3
Laszlog	RSB	round	3
Menecerf	RSB	round	3
Qualine	RSB	flat	3
Nouba	RSB	hollow	3
Magie	RSB	round	3
Saphira	RSB	flat	3
Gorgona	RSB	hollow	3
Ratina	RSB	flat	3
La plus	RSB	round	3
Iraty	RSB	flat	3
Saouré	RSB	flat	2,5

		Percentage of time spent ears in the following positions in stall			
**Individual**	**Population**	**forwards**	**sidewards**	**backwards**	**asymetrical**
Bul	NC	NA	NA	NA	NA
Bounty	NC	NA	NA	NA	72
Momo	NC	NA	NA	NA	NA
Pepito	NC	NA	NA	NA	NA
Haribo	NC	NA	NA	NA	NA
Kansou	NC	NA	NA	NA	NA
Julie	NC	NA	NA	NA	NA
Whinney	NC	NA	NA	NA	NA
Quassie	NC	NA	NA	NA	NA
Hookah	NC	NA	NA	NA	NA
Paloma	NC	NA	NA	NA	NA
Cooper	RSA	0,00	60,00	16,00	24,00
Artiste	RSA	54,55	36,36	9,09	0,00
Man	RSA	16,67	83,33	0,00	0,00
Talisman	RSA	20,00	45,00	35,00	0,00
Centurion	RSA	0,00	22,22	66,67	11,11
Quartz	RSA	7,69	23,08	61,54	7,69
Orion	RSA	7,69	30,77	61,54	0,00
Stevens	RSA	3,57	39,29	57,14	0,00
Ram Dam	RSA	4,55	77,27	18,18	0,00
Lady	RSA	18,52	66,67	7,41	7,41
Quaïnah	RSA	20,00	73,33	0,00	6,67
Taka	RSA	3,33	93,33	3,33	0,00
One girl	RSA	0,00	33,33	66,67	0,00
Palo	RSA	0,00	10,53	89,47	0,00
Royal	RSA	18,18	22,73	50,00	9,09
Nectar	RSA	0,00	71,43	28,57	0,00
Ricky	RSA	20,00	73,33	6,67	0,00
Quitto	RSA	8,33	75,00	8,33	8,33
Sisi	RSA	5,26	73,68	21,05	0,00
Phaistos	RSB	13,33	80,00	6,67	0,00
Asterix	RSB	14,29	85,71	0,00	0,00
Balzan	RSB	16,67	83,33	0,00	0,00
Ty time	RSB	9,52	66,67	19,05	4,76
Manu	RSB	9,52	90,48	0,00	0,00
Tempo	RSB	60,00	40,00	0,00	0,00
Loft	RSB	4,55	72,73	13,64	9,09
Laszlog	RSB	66,67	33,33	0,00	0,00
Menecerf	RSB	37,50	62,50	0,00	0,00
Qualine	RSB	5,88	64,71	29,41	0,00
Nouba	RSB	17,86	82,14	0,00	0,00
Magie	RSB	20,00	60,00	6,67	13,33
Saphira	RSB	48,00	52,00	0,00	0,00
Gorgona	RSB	16,00	36,00	41,67	8,00
Ratina	RSB	0,00	96,00	4,00	0,00
La plus	RSB	11,11	77,78	11,11	0,00
Iraty	RSB	0,00	48,00	40,00	12,00
Saouré	RSB	20,00	60,00	20,00	0,00

		Percentage of time spent ears in the following positions in pasture			
**Individual**	**Population**	**forwards**	**sidewards**	**backwards**	**asymetrical**
Bul	NC	0,00	100,00	0,00	0,00
Bounty	NC	21,43	78,57	0,00	0,00
Momo	NC	66,67	33,33	0,00	0,00
Pepito	NC	7,41	92,59	0,00	0,00
Haribo	NC	25,00	68,75	0,00	6,25
Kansou	NC	8,00	92,00	0,00	0,00
Julie	NC	43,48	56,52	0,00	0,00
Whinney	NC	37,50	62,50	0,00	0,00
Quassie	NC	33,33	66,67	0,00	0,00
Hookah	NC	20,69	79,31	0,00	0,00
Paloma	NC	33,33	66,67	0,00	0,00
Cooper	RSA	45,16	51,61	0,00	3,23
Artiste	RSA	41,94	54,84	3,23	0,00
Man	RSA	41,94	48,39	3,23	6,45
Talisman	RSA	40,61	36,06	23,33	0,00
Centurion	RSA	67,74	29,03	3,23	0,00
Quartz	RSA	38,71	58,06	0,00	3,23
Orion	RSA	61,29	35,48	3,23	0,00
Stevens	RSA	64,52	29,03	6,45	0,00
Ram Dam	RSA	66,01	16,13	17,86	0,00
Lady	RSA	16,67	33,33	16,67	33,33
Quaïnah	RSA	58,06	41,94	0,00	0,00
Taka	RSA	54,84	45,16	0,00	0,00
One girl	RSA	61,29	35,48	3,23	0,00
Palo	RSA	39,62	18,71	41,67	0,00
Royal	RSA	51,61	48,39	0,00	0,00
Nectar	RSA	54,84	45,16	0,00	0,00
Ricky	RSA	51,61	48,39	0,00	0,00
Quitto	RSA	70,97	29,03	0,00	0,00
Sisi	RSA	19,35	80,65	0,00	0,00
Phaistos	RSB	45,16	51,61	0,00	3,23
Asterix	RSB	61,29	38,71	0,00	0,00
Balzan	RSB	61,19	35,48	3,33	0,00
Ty time	RSB	81,05	6,45	12,50	0,00
Manu	RSB	48,39	51,61	0,00	0,00
Tempo	RSB	45,16	54,84	0,00	0,00
Loft	RSB	61,29	38,71	0,00	0,00
Laszlog	RSB	32,26	58,06	6,45	3,23
Menecerf	RSB	NA	NA	NA	NA
Qualine	RSB	16,13	74,19	0,00	9,68
Nouba	RSB	80,65	19,35	0,00	0,00
Magie	RSB	6,45	87,10	6,45	0,00
Saphira	RSB	45,16	54,84	0,00	0,00
Gorgona	RSB	29,03	70,97	0,00	0,00
Ratina	RSB	54,84	45,16	0,00	0,00
La plus	RSB	25,81	6,45	64,52	3,23
Iraty	RSB	64,52	35,48	0,00	0,00
Saouré	RSB	38,71	54,84	0,00	6,45

Individual	Population	Percentage of time spent facing the wall	SB/ARB occurrences	Number of aggressive behaviours
Bul	NC	NA	0	0
Bounty	NC	NA	0	1
Momo	NC	NA	0	0
Pepito	NC	NA	0	0
Haribo	NC	NA	0	0
Kansou	NC	NA	0	0
Julie	NC	NA	0	0
Whinney	NC	NA	0	0
Quassie	NC	NA	0	0
Hookah	NC	NA	0	0
Paloma	NC	NA	0	0
Cooper	RSA	6,45	0	1
Artiste	RSA	9,68	1	0
Man	RSA	0,00	2	0
Talisman	RSA	0,00	2	2
Centurion	RSA	0,00	10	2
Quartz	RSA	6,45	19	6
Orion	RSA	0,00	25	0
Stevens	RSA	0,00	0	0
Ram Dam	RSA	6,45	8	1
Lady	RSA	16,13	52	4
Quaïnah	RSA	0,00	0	0
Taka	RSA	22,58	8	0
One girl	RSA	0,00	10	2
Palo	RSA	0,00	0	4
Royal	RSA	3,23	19	1
Nectar	RSA	3,23	3	0
Ricky	RSA	3,23	0	2
Quitto	RSA	0,00	0	1
Sisi	RSA	0,00	0	6
Phaistos	RSB	0,00	5	0
Asterix	RSB	0,00	0	0
Balzan	RSB	3,23	6	0
Ty time	RSB	0,00	0	0
Manu	RSB	0,00	0	0
Tempo	RSB	9,68	0	0
Loft	RSB	3,23	0	4
Laszlog	RSB	6,45	7	4
Menecerf	RSB	0,00	29	0
Qualine	RSB	0,00	14	3
Nouba	RSB	0,00	0	1
Magie	RSB	0,00	0	0
Saphira	RSB	3,23	0	2
Gorgona	RSB	0,00	10	0
Ratina	RSB	0,00	10	0
La plus	RSB	0,00	0	0
Iraty	RSB	3,23	7	0
Saouré	RSB	6,45	28	1

Individual	Population	TCSS1	TCSS RS scale (stall)	TCSS RS scale (pasture)
Bul	NC	28,5	NA	9
Bounty	NC	46	NA	18
Momo	NC	28,5	NA	31,88
Pepito	NC	28,5	NA	5,6
Haribo	NC	28,5	NA	2,4
Kansou	NC	28,5	NA	4
Julie	NC	28,5	NA	12
Whinney	NC	28,5	NA	15
Quassie	NC	28,5	NA	10,8
Hookah	NC	28,5	NA	13,2
Paloma	NC	28,5	NA	20
Cooper	RSA	46	5,33	1,85
Artiste	RSA	42,5	6	4
Man	RSA	44	10,8	5,54
Talisman	RSA	67,5	1,2	4,5
Centurion	RSA	77,5	0	7,2
Quartz	RSA	90	1	6
Orion	RSA	59,5	13,5	8,31
Stevens	RSA	28,5	1,71	4
Ram Dam	RSA	68,5	5,33	1,6
Lady	RSA	28,5	3,6	3,27
Quaïnah	RSA	51	0	4,24
Taka	RSA	77,5	3,6	1,5
One girl	RSA	90,5	0	8,47
Palo	RSA	57,5	0	1,5
Royal	RSA	75,5	6	5,25
Nectar	RSA	45,5	3,43	4
Ricky	RSA	52	6,86	6,75
Quitto	RSA	46	2,4	11,29
Sisi	RSA	60,5	1,33	3,6
Phaistos	RSB	46,5	9,333333333	11,07692308
Asterix	RSB	28,5	0	8,25
Balzan	RSB	47,5	0	11,25
Ty time	RSB	28,5	10,28571429	12,75
Manu	RSB	28,5	6	8,25
Tempo	RSB	28,5	5,454545455	19,2
Loft	RSB	57,5	4	11,25
Laszlog	RSB	78	1,5	5,142857143
Menecerf	RSB	NA	6,461538462	NA
Qualine	RSB	83	1,333333333	6,461538462
Nouba	RSB	46	6	8,210526316
Magie	RSB	28,5	3,428571429	22,28571429
Saphira	RSB	52	0	2,25
Gorgona	RSB	54	2,4	7,578947368
Ratina	RSB	54	7,2	6,315789474
La plus	RSB	28,5	9,230769231	12
Iraty	RSB	49	12	5,052631579
Saouré	RSB	78	0	1,846153846

Individual	Population	TCSS population scale (stall)	TCSS population scale (pasture)
Bul	NC	NA	11,5
Bounty	NC	NA	17
Momo	NC	NA	11,5
Pepito	NC	NA	11,5
Haribo	NC	NA	11,5
Kansou	NC	NA	11,5
Julie	NC	NA	11,5
Whinney	NC	NA	11,5
Quassie	NC	NA	11,5
Hookah	NC	NA	11,5
Paloma	NC	NA	11,5
Cooper	RSA	36,5	35
Artiste	RSA	36	44
Man	RSA	20,5	34
Talisman	RSA	40,5	35
Centurion	RSA	51	48,5
Quartz	RSA	65,5	56,5
Orion	RSA	43	42,5
Stevens	RSA	27,5	31,5
Ram Dam	RSA	46	43,5
Lady	RSA	58,5	72,5
Quaïnah	RSA	15	20
Taka	RSA	38,5	42
One girl	RSA	51	48,5
Palo	RSA	45	32,5
Royal	RSA	51	44,5
Nectar	RSA	38	33,5
Ricky	RSA	33,5	36
Quitto	RSA	25	25,5
Sisi	RSA	38	34,5
Phaistos	RSB	44,6	41,6
Asterix	RSB	26,1	29,1
Balzan	RSB	46,5	49,5
Ty time	RSB	35,6	29,1
Manu	RSB	26,1	29,1
Tempo	RSB	37,5	40,5
Loft	RSB	52,5	47
Laszlog	RSB	62	73
Menecerf	RSB	53,6	NA
Qualine	RSB	69,6	61,1
Nouba	RSB	33,1	28,6
Magie	RSB	32,1	37,1
Saphira	RSB	41,5	44,5
Gorgona	RSB	59,6	49,1
Ratina	RSB	50,6	49,1
La plus	RSB	33,6	38,6
Iraty	RSB	60,5	51
Saouré	RSB	80	72,5
